# Telemedicine for Adults With Cochlear Implants in the United Kingdom (CHOICE): Protocol for a Prospective Interventional Multisite Study

**DOI:** 10.2196/27207

**Published:** 2022-04-13

**Authors:** Helen Cullington, Padraig Kitterick, Philippa Darnton, Tracy Finch, Kate Greenwell, Carol Riggs, Mark Weal, Dawn-Marie Walker, Andrew Sibley

**Affiliations:** 1 University of Southampton Auditory Implant Service University of Southampton Southampton United Kingdom; 2 Nottingham Hearing Biomedical Research Unit National Institute for Health Research Nottingham United Kingdom; 3 Wessex Academic Health Science Network Southampton United Kingdom; 4 Northumbria University Newcastle upon Tyne United Kingdom; 5 Centre for Clinical and Community Applications of Health Psychology University of Southampton Southampton United Kingdom; 6 School of Electronics and Computer Science University of Southampton Southampton United Kingdom; 7 School of Health Sciences University of Southampton Southampton United Kingdom

**Keywords:** cochlear implants, hearing, deafness, telemedicine, patient-centered care

## Abstract

**Background:**

Cochlear implants provide hearing to approximately 750,000 people with deafness worldwide; these patients require lifelong follow-up. Care for adults with implants in the United Kingdom occurs at one of 19 centers, which may be far from the patients’ homes. In a previous randomized controlled trial, we successfully introduced person-centered care. We designed, implemented, and evaluated the following remote care pathway: a personalized web-based support tool, home hearing check, self–device adjustment, and upgrading of sound processors at home rather than in the clinic. The remote care group had a significant increase in empowerment after using the tools, and the patients and clinicians were keen to continue. We would now like to scale up these improvements as an option for >12,000 UK adults using implants; we are commissioning an independent evaluation of this intervention and rollout to establish if it achieves its aims of more empowered and confident patients; more accessible and equitable care; stable hearing; more efficient, person-centered, and scalable service; and more satisfied and engaged patients and clinicians.

**Objective:**

This study aims to evaluate the impact and rollout of a person-centered clinical care pathway via telemedicine for adults with cochlear implants in the United Kingdom, using both outcomes and process evaluation.

**Methods:**

This project will scale up and evaluate a person-centered long-term follow-up pathway for adults using cochlear implants through a personalized website, including a home hearing check, uploading photos of cochlear implant site, listening in noise and music practice, ordering of spares, questionnaires, and other resources. Both quantitative and qualitative analyses will be conducted, and they will be both an outcome and process evaluation.

**Results:**

As of July 2021, the trial is closed, and all data collection is complete. The evaluation report is expected to be published in December 2021, and the research data have not yet been analyzed.

**Conclusions:**

This project will present the results of the first scaling up of a remote care pathway for adults with cochlear implants in the United Kingdom.

**Trial Registration:**

International Standard Randomized Controlled Trial Number ISRCTN51668922; https://www.isrctn.com/ISRCTN51668922

**International Registered Report Identifier (IRRID):**

DERR1-10.2196/27207

## Introduction

### Background

Cochlear implants are the most successful of all neural prostheses [1]; they can provide hearing to people with severe to profound deafness. Approximately 1600 people receive cochlear implants in the United Kingdom (UK) each year [2]. The total number of people with implants is approximately 20,000 in the UK (estimated from [2]) and approximately 0.75 million worldwide (estimated from [3-5]). Numbers are likely to increase rapidly, with only approximately 5% of eligible people in the UK and worldwide having received an implant [4,6]. The number of people of retirement age is projected to increase by 28% by 2035 [7], indicating a further increase in the number of people with hearing impairment. Adult cochlear implant care in the United Kingdom is provided at one of 19 tertiary centers involving assessment, surgery, and a resource-intensive acute phase of device adjustment and rehabilitation. When a patient attends a long-term follow-up appointment, the following tasks may be performed: speech recognition testing, device adjustment, rehabilitation, equipment check and troubleshooting, and the provision of replacement or upgraded equipment. Currently, UK implant centers review patients on a clinic-led schedule, which means review appointments that provide little benefit to the patient can occur. Conversely, when some patients attend routine appointments, there is hearing deterioration that the patient had not noticed. This is often remedied by replacing equipment, which the patient could have done at home.

Cochlear implant centers may be several hours away from the patient’s home, necessitating travel expenses, time off work, and family disruption; distance to care is a significant barrier to hearing care worldwide [8]. Making this care pathway person centered instead may provide a more efficient and effective service and allow more timely identification of issues; evidence suggests that person-centered care can improve a range of factors, including patient experience, care quality, and health outcomes, and may help clinics manage a growing number of people with long-term conditions [9].

We previously designed and implemented a remote care pathway for adults with cochlear implants to enable them to perform some of the follow-up tasks themselves at home. We ran a 6-month clinical trial with 60 people randomized to either a telemedicine remote care pathway or a control group who followed their usual appointment schedule [10]. The main outcome evaluated was patient empowerment, which has been shown to be strongly linked to better outcomes in people with long-term conditions. We found that only the remote care group had a significant increase in their cochlear implant empowerment after using remote care tools. The quality of life remained unchanged in the 2 groups. The hearing check results in the clinic improved in the remote care group, although they did not notice a change. However, the control group felt that their hearing had become slightly worse. This may suggest that the remote care group was better able to take action to keep their hearing stable during the trial, or, perhaps, the control group felt they were missing out on a desirable opportunity to take a more active role in their hearing health care.

Discontinuing routine appointments and attending the clinic only when there is a clinical need may provide the following benefits for patients using cochlear implants:

- More stable hearing (problems identified and resolved quicker)
- Better hearing (ability to fine-tune when away from the clinic)
- Convenience of not traveling for routine appointments
- Reduction of travel costs and time, time off work, and disruption to family life
- Increased confidence in managing one’s own hearing
- Greater equality in service delivery (same level of service regardless of distance from the clinic)

It may also mean that the clinic has greater resources (time, money, and space) to see both patients with more complex needs and an expanding population of new patients. People using cochlear implants and their families generally like to take a more active role in their care and welcome the use of technology to assist self-care [11,12]. The National Health Service (NHS) has a strong commitment to promoting self-care and self-management [13] for people with long-term conditions [14], with “the vision of a citizen-centred, digitally-enabled, health and social care system” [15]. Evidence shows a significant improvement in outcomes when patients use self-management tools [16], and those who are activated and involved in their care tend to have better health outcomes [17,18]. We are now ready to scale up successful remote care interventions for many more people with cochlear implants in the United Kingdom.

### Objective

This study aims to evaluate the impact and rollout of a person-centered clinical care pathway via telemedicine for adults with cochlear implants in the United Kingdom, using both outcomes and process evaluation.

## Methods

### Project Design and Setting

This is a prospective, interventional, multisite, quality improvement project led and sponsored by the University of Southampton. All research measures will be self-administered on the web or by a paper questionnaire at the patient’s home or other locations of their choice. The staff will complete the measures at work or at a location of their choice. Data collection began when the first site opened on June 11, 2019**,** and continued until January 31, 2021. Clinics will join the study when appropriate local approvals are obtained; therefore, it is likely that the follow-up at each clinic will be for different durations.

### Intervention

**Figure 1:**
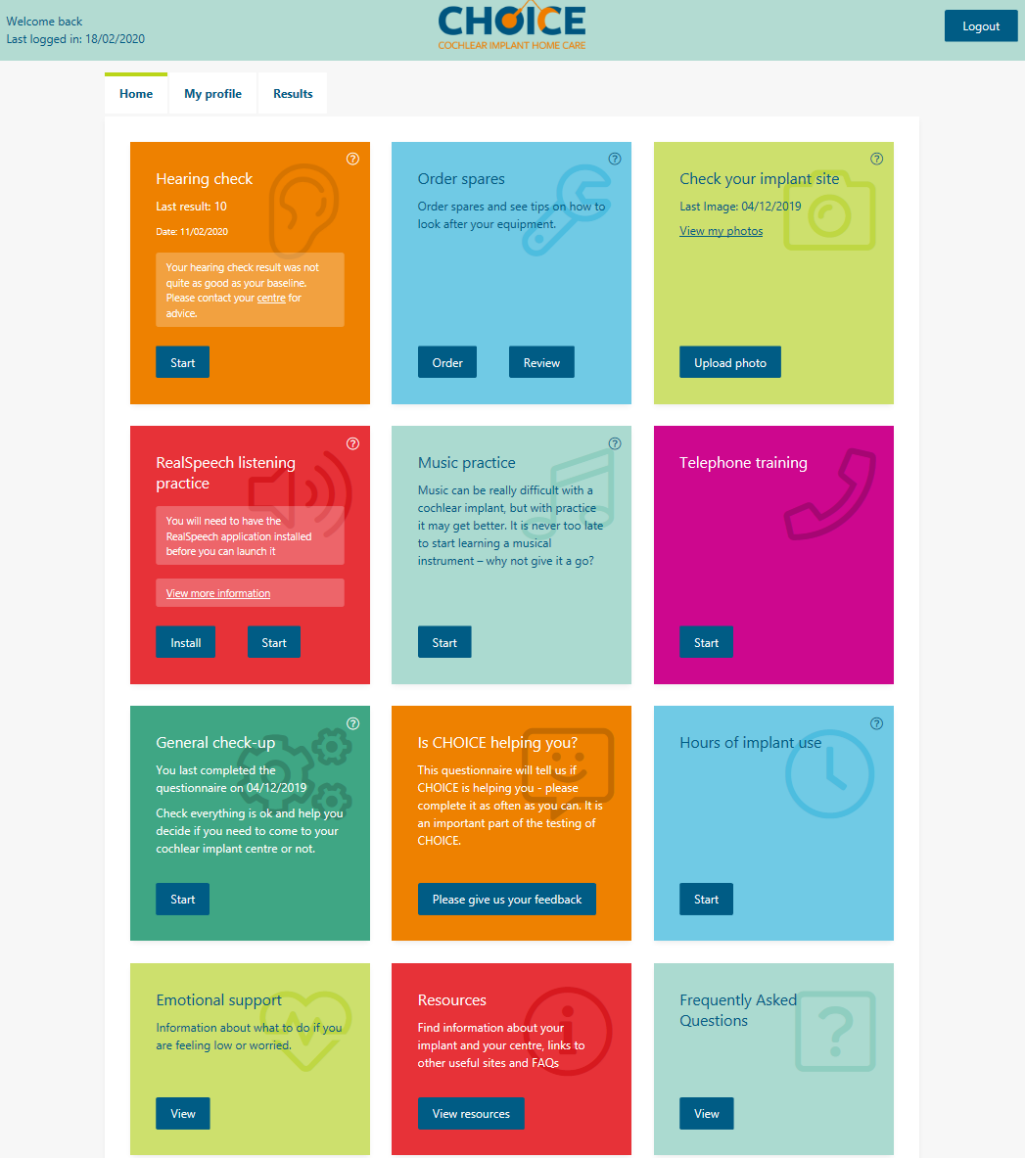
Cochlear implant home care (CHOICE) web app patient dashboard.

This project introduced a remote care pathway option for adults using cochlear implants: cochlear implant home care (CHOICE). We built a personalized, scalable, and responsive web app (not a native application but accessible from any internet browser) based on our previously trialed CIRCA (Cochlear Implant Remote Care website; built in LifeGuide [19]). The app incorporated a home hearing check based on the triple digit test [20], personalized reminders (eg, change microphone cover), rehabilitation exercises (listening in noise, music, and telephone practice), uploading a photo of the cochlear implant surgery site (behind the ear) for review by the clinical care team, information and training, logging the number of hours patients used their cochlear implant (optional and self-reported only), evaluation measures, ordering replacement parts for their cochlear implant, emotional support resources, and questionnaires (Figure 1). The home hearing check provides a screen for whether the patient should come to the clinic based on comparison with a baseline check. Speech perception in noise testing using spoken digits (eg, *one*) has the advantage of digits being highly familiar stimuli usually known by people with even limited language skills. Digit testing requires a closed, set response and is, thus, suitable for self-testing over the telephone or internet [21,22] and has a minimal learning effect [23]. The test correlates well with speech recognition in noise with sentences in people using cochlear implants [24-27].

It is vital that patients remain vigilant in preventing medical issues related to their cochlear implants. This mainly involves appropriate action for ear infections (following the center’s protocol) and checking the site of the implant and skin under the coil magnet. The CHOICE website advises patients to contact their clinical care center with any medical concerns. The web app has the functionality to upload and store photos of the patient’s implant site. Patients will be asked to take a baseline photograph at an early stage to provide a comparison with later images.

The patient’s clinician at their cochlear implant center will have access to their results and web app use in the CHOICE web-based clinician dashboard. Cochlear implant center clinic appointments will be given if required, requested, or indicated by the outputs of the remote care tools. Otherwise, the patients in this pathway will continue with remote care. Participants may access the web app tools as often as they wish.

Automated flagging by email and website notifications will be the cornerstone of the remote care pathway. This will ensure that the patient’s problems are not missed and will provide the most efficient use of clinician time. Some patient flagging situations are as follows: no interaction with CHOICE for 3 months; hearing deterioration; patients who indicate that they need help on the general check-up questionnaire; each time a photo is uploaded, clinicians need to review it; replacement stock items are required; patient reports their daily sound processor use is **<**6 hours; request to leave CHOICE; and freedom of information request.

When an alert is received, the patient’s clinician will decide whether further action is required; for example, an in-center appointment.

The CHOICE website conforms to the following specifications: risk management (ISO 14971:2007) and software life cycle (BS EN 62304:2006) and complies with the requirements of the European Union directive 93/42/ECC for medical devices. It is Conformitè Europëenne marked and registered with the Medicines and Healthcare products Regulatory Agency as a Class 1 medical device.

### Participants

#### Overview

The following 7 UK centers will offer CHOICE to their patients:

1. St Thomas’ Hospital Hearing Implant Center, London
2. University of Southampton Auditory Implant Service
3. Royal National Throat Nose and Ear Hospital, London
4. Nottingham Auditory Implant Program
5. North East Regional Cochlear Implant Program, Middlesbrough
6. The Richard Ramsden Center for Hearing Implants, Manchester
7. Emmeline Center, Cambridge

All adult sites were contacted about CHOICE and its evaluation; these sites wanted to be involved. CHOICE is currently an intervention for adults only. Initially, scaling up to only 7 of the adult sites will allow for detailed evaluation. Depending on the evaluation results, CHOICE may be offered to all sites in the future.

#### Proposed Sample Size

We do not yet know what proportion of patients will choose to follow this pathway, as the previous study was a single-center randomized controlled trial involving a limited number of patients [10]. However, 7 centers care for approximately one-third of the approximately 12,000 adults [28] with cochlear implants in the United Kingdom. At the early stages of project planning, we estimated that if 40% of patients enrolled, this may involve approximately 1700 patients. We expect this to be the upper limit for recruitment. Scaling up a digital health tool for people with cochlear implants has not been done before; thus, we cannot predict the uptake. We anticipate that up to 10 members of staff per will be involved per site (total 70). As the aim of the project was not to formally test a hypothesis, a sample size calculation was not conducted.

#### Recruitment

We recommend shared decision-making among the patient, their family, and their clinician to decide who should be on a remote care pathway [29]. Factors that need to be considered include the patient’s care needs; routine maintenance of equipment; access to technology; mobility; literacy; dexterity; any comorbidities (eg, visual impairment); and other factors, such as do they live alone and do they have transport. All patients who meet the inclusion criteria and, after discussing with their clinician as needed, choose the remote care pathway will be invited to participate in the study. Only those who consent to the study will be able to continue with remote care at this stage. As patients’ circumstances and abilities change, we recommend service delivery flexibility, with easy transfer to a clinic-based care model, if required. Staff at participating centers will be invited to take part and sign a consent form for their data to be included in the evaluation. Participant recruitment commenced on June 11**,** 2019, and continued until January 31, 2021.

#### Patient Inclusion Criteria

The inclusion criteria for patients are as follows:

1. Using a cochlear implant (any device—unilateral or bilateral)
2. Living in the United Kingdom
3. Aged ≥18 years
4. Able to give informed consent to data sharing
5. Access to a computer or device with internet access
6. Willing and able to comply with CHOICE pathway
7. Willing and able to comply with the evaluation

#### Health Professional Inclusion Criterion

The inclusion criterion for health professionals was being a staff member at participating cochlear implant centers.

### Evaluation and Research Outcomes

#### Overview

The Wessex Academic Health Science Network (AHSN) will perform an independent evaluation to assess the impact and success of the care pathway on patients, staff, and services and understand the process of implementing CHOICE using a concurrent triangulation mixed methods design. It will be both an outcome and process evaluation. The evaluation was commissioned in September 2017 and is informed by a growing research base on the challenges associated with the adoption and spread of digital programs. The research team will collect the clinical outcome measures. All the outcomes, methods, and measures corresponding to the research questions are listed in Table 1.

**Table 1:** Outcomes, methods, and measures collected from patients, staff, and services.

Outcome			Research question		Method		Measures		Time point
**Patients**									
	Patient impact (engagement)	1		Quantitative		CHOICE^a^ web app data: number of log-ins, time spent on CHOICE, uses of self–device adjustment (if appropriate), and uses of home hearing check		All data	
	Patient impact (quality)	1		Quantitative		Number of errors in CHOICE, adverse events, and missed issues		All data	
	Patient impact	1		Quantitative		Survey about use of follow-up care (consequences for travel cost, time, hours off work, and child care [including accompanying person])		Once at patient focus group or interview	
	Patient impact	1		Quantitative		NHS^b^ Friends and Family Test		Minimum of twice: baseline (on registration) and after using CHOICE for several months	
	Patient impact	1		Qualitative		Focus groups		Once: planned but unable to happen because of the COVID-19 pandemic	
	Patient impact	1		Qualitative		One-on-one interviews		Once: for patients who prefer one-on-one interviews or if focus groups cannot occur; toward the end of the project	
	Patient empowerment	2		Quantitative		PAM^c^ questionnaire and CI-EMP^d^ questionnaire		Baseline (on registration) and 6 months following registration or end of the project—whichever comes sooner	
	Patient hearing	2		Quantitative		Home hearing check results		All data	
	Patient change in empowerment, hearing, and quality of life	2		Quantitative		Global ratings of change questionnaire		Baseline (on registration) and 6 months following registration or end of the project—whichever comes sooner	
	Patient health-related quality of life, including hearing	2		Quantitative		HUI3^e^ questionnaire		Baseline (on registration) and 6 months following registration or end of the project—whichever comes sooner	
	Patient health-related quality of life	2		Quantitative		EQ-5D-5L^f^ questionnaire		Baseline (on registration) and 6 months following registration or end of the project—whichever comes sooner	
	Patient preference of service delivery	2		Quantitative		Discrete Choice Experiment questionnaire		Baseline (on registration) and 6 months following registration or end of the project—whichever comes sooner	
	Patient confidence and experience	4 and 5		Quantitative		R-Outcomes surveys		Baseline (on registration) and every 6 months; some participants may choose to complete an optional, shorter questions set more often	
**Staff**									
	Staff impact (engagement)	1		Quantitative		CHOICE web app data from clinician dashboard: number and type of log-ins		All data	
	Staff impact	1		Quantitative		NHS Friends and Family Test		Minimum of twice: baseline (on registration) and after using CHOICE for several months	
	Staff behavior	1		Quantitative		NoMAD^g^ questionnaire		At interview or by email and by email request toward the end of the evaluation	
	Staff impact	1 and 9		Qualitative		Focus groups (staff)		Once: planned but unable to happen because of the COVID-19 pandemic	
	Staff impact	1 and 9		Qualitative		One-on-one interviews (staff)		Once for key staff who are not available for the on-site focus group; toward the end of the project	
	Staff experience	7		Quantitative		R-Outcomes surveys		Baseline (on registration) and every 6 months	
**Services**									
	Spread, equity of access, and resource use	3, 4, and 8		Quantitative		Clinic activity information		See Multimedia Appendix 1 for more details	

^a^CHOICE: cochlear implant home care.

^b^NHS: National Health Service.

^c^PAM: Patient Activation Measure.

^d^CI-EMP: Cochlear Implant Empowerment Scale.

^e^EQ-5D-5L: EuroQol 5-Dimension 5-Level.

^f^HUI3: Health Utilities Index Mark 3.

^g^NoMAD: Normalization Measure Development.

#### Primary Research Questions

This study attempts to answer the following primary research questions:

1. Evaluation: What is the impact of the rollout of the new care pathway on users of the program (people with cochlear implants and the staff)?
2. Research: Does the new care pathway increase empowerment for people with cochlear implants while having no detrimental effect on their hearing and quality of life?

#### Secondary Research Questions

This study attempts to answer the secondary research questions provided in Textbox 1.

**Secondary research questions.**

3. What is the extent of the spread of the new care pathway?

- What has facilitated the adoption of the new care pathway?
- What has hindered the adoption of the new care pathway?

4. Does the new care model improve patients’ confidence to self-manage their cochlear implant as measured by patient-reported outcomes of health confidence, health status, and personal well-being?

- Do patients initiate review appointments with the service rather than rely on or wait for appointments scheduled by the service?

5. Does the new care model improve patients’ experience of follow-up care?

- Do patients engage with the technology as measured by patient-reported outcomes of digital confidence and perceived value of the tool?

6. Does the new care model improve equity of access to follow-up care?

7. Does the new model of care improve the experience of staff working in the service, as measured by staff-reported outcomes of job confidence and work well-being?

- Do staff have confidence in the new care model, as measured by staff-reported outcomes of digital confidence and perceived value of the tool?
- Do they recommend it?

8. Does the new care model improve the use of resources by reducing the need for follow-up appointments and enabling the service to be delivered by a different skill mix?

9. What lessons can be learned from the implementation process that will benefit the spread and adoption of this model?

#### Patient Outcomes

##### Quantitative Measures

All data will be downloaded from the CHOICE web app, and patient use of all elements of CHOICE, including the hearing check, will be assessed. Errors in CHOICE, adverse events, and missed patient issues will be collected during the study period. Patients who take part in the focus group or interview will be asked to complete a short survey about the cost implications of switching to remote care (eg, impact on travel costs and need for childcare).

Quantitative data about patients’ use of CHOICE will be collected using the R-Outcomes survey tool [30]. These measures share a common framework with 4 items and 4 responses suitable for use on a mobile device and are validated, short, and have a lower reading age than other measures. R-Outcomes are incorporated into CHOICE and will assess the patients’ health, well-being, health confidence, digital readiness, and user experience. The NHS Friends and Family Test was also incorporated into the CHOICE web app, asking the question, “How likely are you to recommend this service to friends and family if they need similar care or treatment?” with 6 response options ranging from *extremely likely* to *extremely unlikely* [31].

We will use the following measures to assess empowerment, health-related quality of life, hearing, and patient care pathway preference: Patient Activation Measure (PAM), Cochlear Implant Empowerment Scale (CI-EMP), EuroQoL 5-Dimension 5-Level (EQ-5D-5L) questionnaire, Health Utilities Index Mark 3 (HUI3), a global change rating, and a discrete choice experiment (DCE). PAM is a well-validated generic measure of patient activation that evaluates the knowledge, skills, beliefs, and behaviors that patients have for self-management of their long-term condition [32,33]. The CI-EMP is a questionnaire specifically designed to measure how empowered people are to manage their own cochlear implant care [34]. The EQ-5D-5L is a standardized health outcome measure comprising five dimensions: mobility, self-care, usual activities, pain or discomfort, and anxiety or depression [35]. The HUI3 is a multi-attribute health status classification system that evaluates eight domains: vision, hearing, speech, ambulation, dexterity, emotion, cognition, and pain [36].

The global rating of change scales will be used to capture whether patients perceive a change in their hearing, empowerment, and quality of life to determine whether any changes observed in the PAM, CI-EMP, HUI3, or EQ-5D-5L are meaningful; that is, whether they were perceived by patients.

**Figure 2:**
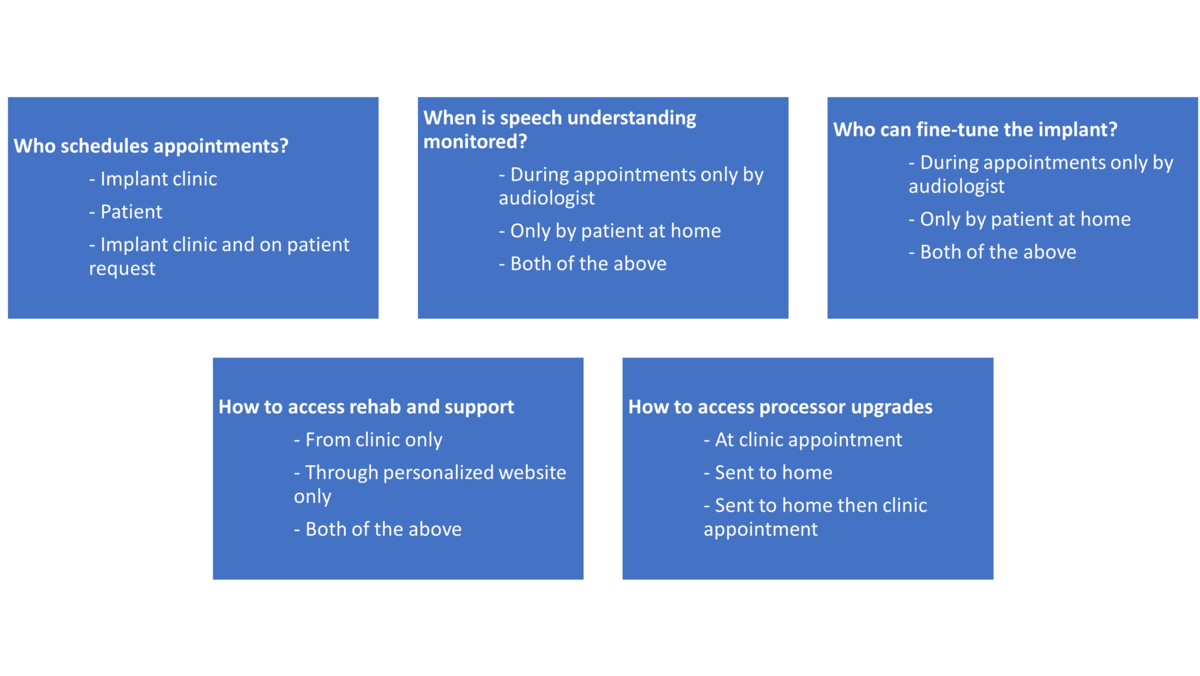
The 5 elements of the discrete choice experiment.

We designed a DCE to assess the effects of the following five care pathway attributes on the preferences of the participants for remote care (Figure 2):

1. Who decides when the next clinic appointment will be?
2. When is the ability to understand speech monitored?
3. Who can fine-tune the cochlear implant?
4. Where can patients get rehabilitation and troubleshooting information that is personalized to their needs?
5. How are upgrades to sound processors provided?

Each attribute had 3 levels that described different approaches and degrees of remote care; for example, the choices for who decides when the next clinic appointment will be *the implant clinic*, *the patient,* or *the implant clinic (however, the patient can request appointments when required)*. The experiment was constructed using the mix and match design method [37], as implemented in the *support.CEs* package for the R statistical environment [38]. The experimental design was organized into 2 blocks to reduce the number of questions each participant had to complete, and patients will be randomly assigned to complete either block 1 or block 2. The design requirements of 5 attributes per alternative, 2 alternatives per choice question, and 2 experimental blocks resulted in the allocation of 9 discrete choices per block. The role of the DCE is to help us learn about how the different elements of the care pathway interact to shape participant preferences for remote care compared with the usual pathway. It is possible that the preferences that patients have for remote care could relate to their outcomes, and we will explore these relationships using exploratory correlational analyses.

##### Qualitative Measures

Although the qualitative fieldwork was initially planned as focus groups of patients and staff at each site, because of the COVID-19 pandemic, this changed to telephone or web-based interviews. Up to 20 patients per site will be recruited. The interviews will be audio recorded, transcribed, and managed using NVivo (version 12; QSR International); 2 Wessex AHSN qualitative evaluators will conduct all interviews.

#### Staff Outcomes

##### Quantitative Measures

CHOICE web app data about staff use of the clinician dashboard will be downloaded and analyzed. The staff will also be asked to complete the NHS Friends and Family Test and R-Outcomes (with additional measures of work well-being and innovation adoption) within the CHOICE clinician dashboard.

##### Qualitative Measures

As for patients, focus groups were planned but changed to telephone or web-based interviews because of the COVID-19 pandemic; up to 10 staff per site will be recruited for one-on-one interviews.

##### Services Outcomes: Quantitative Measures

Local service-level activity data will be collected at all sites with a view to assessing resource use and the workforce (Multimedia Appendix 1). We aim to obtain data from all clinic patients to maximize the sample size. A cohort of patients will also be identified for comparison. This will comprise patients registered with clinics but who have not yet been offered the new care model. We will look at the aggregated clinical activity before and after the introduction of the tool (eg, numbers of outpatient appointments and DNAs in the inclusion group). There will not be a control group of patients undergoing the same measures as the intervention group. We will also analyze the centers’ previously collected service-level data to evaluate the current pathway.

#### Process Evaluation

This part of the evaluation will assess what lessons can be learned from the implementation process and what *key ingredients* are replicable to other clinical settings.

##### Evaluation of the Behaviors of Staff Involved in Implementation of CHOICE

The evaluation design is informed by the Normalization Process Theory [39], which provides a pragmatic framework for collecting and analyzing what the staff does in response to changes in the model of care, and the nonadoption, abandonment, scale-up, spread, and sustainability (NASSS) framework [40] will inform the design of the staff interviews. In addition, the Normalization Measure Development questionnaire [41] will be administered to the staff early on in the rollout and later by email at the end of the data collection period.

##### Evaluation of the Factors That Have Facilitated or Hindered the Adoption of CHOICE

An analysis of the findings from the qualitative data sources will be compared with factors known to be important for spread and adoption [40]. This will enable us to understand the factors that facilitate or inhibit the embedding of CHOICE in the care pathway.

##### Assessment of Resource Use and Workforce

As this model is scaled up, it will offer important learnings on how it can be delivered most efficiently and whether the anticipated changes in clinic activity and type (as a consequence of remote care options) have any implications for the clinic workforce. For example, if the reason for clinic attendance is known in advance, as it is requested by the patient, the patient may not need to be seen by a senior audiologist. Data on the workforce at each site, as well as any changes during the project, will be collected and analyzed.

We will examine the economic impact on the clinic activity of implementing the new care model. We will also apply predictive modeling to understand the impact of scaling up the model beyond a target cohort of several thousand patients. The costs associated with the delivery of follow-up activities will be sourced from each site to understand the impact of uptake of remote care.

### Data Analysis

All data analyses aim to answer the 9 primary and secondary research questions. Statistical analyses will be performed using the SPSS Statistics package (version 26; IBM Corp).

#### Quantitative

Descriptive statistics and graphs will be used to present the data. Data will be displayed visually wherever possible to facilitate sharing with various stakeholders. The significance value will be set at *P*=.05, including Bonferroni corrections for multiple comparisons where appropriate. All repeated-measures data will be compared at baseline and follow-up using analysis of variance to examine any changes in empowerment, hearing, and quality of life in the participants. Surveys will be analyzed at the baseline and follow-up time points using inferential statistical analyses. The choices of participants in the DCE will be subjected to conditional logit model analysis using the *survival* package of the R programming language.

#### Qualitative

The qualitative data from the patient interviews, staff interviews, and case studies will be thematically analyzed separately but brought together in the triangulation phase using synthesis meetings with different involved investigators. To address the evaluation questions, qualitative findings will be synthesized with the quantitative findings. Both theoretical frameworks applied to this evaluation (NASSS and Normalization Process Theory) will be used to facilitate an understanding of the findings. Qualitative interview data will be coded by 2 qualitative evaluators (Wessex AHSN) using a coding framework based on the NASSS framework. A small sample of transcripts will test and refine the framework with an agreement between the coders. The coding framework and coding of transcripts will use NVivo software. Higher order codes and themes will be presented for scrutiny and sensemaking to the wider evaluation team.

#### Missing Data

We anticipate significant missing data because of the large number of outcomes measured and the clinical population. We expect that data will mostly be missing not at random, as those who discontinue the use of CHOICE or drop out are likely to be those who find it less helpful. This may lead to significant bias. There is likely to be a selection bias, as patients who agree to follow a remote care pathway may not be representative of the population. The same will apply to clinicians: those who want to be involved in implementing CHOICE are likely to be more invested in remote care than their colleagues. Following recommendations [42], when data are ready to be analyzed, inspection will suggest whether statistical methods should be used to handle missing data. As this is an outcome and process evaluation, the extent and pattern of missing data will in itself be significant, with nonresponse bias expected. It is also possible that reporting bias may occur; people with cochlear implants are often so grateful for their treatment that they may provide answers in the direction they perceive that the researchers want.

### Monitoring

#### Steering Group

The CHOICE steering group (SG) meets every 4 months and comprises the CHOICE chief investigator; project manager (PM); 2 patients; coordinators of 2 other cochlear implant centers; the lead of the independent evaluation team; and senior representatives from the NHS Specialist Commissioning, The Ear Foundation, and the National Cochlear Implant Users Association. The purpose is to advise and guide the project by reflecting differing stakeholder needs to maximize success and ensure the long**-**term sustainability of the project. The SG acts as a sounding board for the project, particularly in relation to key project risks (including time, cost, quality, commercial, legal, and ethical risks). The SG also deals with safety monitoring, adverse events, data monitoring, deviations from and breaches of protocol, and major project changes.

#### Evaluation Advisory Group

The evaluation advisory group (EAG) is a requirement of the project funder and its remit relates to the independent evaluation of CHOICE. The EAG meets every 3 months and comprises the Wessex AHSN’s Director of Insight (chair), Associate Director of Insight (evaluation lead), program manager, and data analyst; the CHOICE chief investigator and PM; a strategic advisor from Consilium Partners Ltd; the Director of R-Outcomes Ltd; the RUBIS.Qi evaluation lead (coaching organization provided by the funder); and a patient. The CHOICE team does not take decisions on the evaluation but collaborate and provide input as required. The EAG also provides a forum for reflecting on the findings of the evaluation during the course of the project and enable improvements in the scaling up of CHOICE via formative learning.

#### Industry Advisory Group

The industry advisory group was formed to ensure 2-way dialog with the device manufacturers of cochlear implants. This stakeholder group is purposefully separate from the SG so that CHOICE continues its ethos of being patient centric, charity funded, and agnostic of individual industry parties. The industry advisory group meets every 6 months and comprises the chief investigator and PM and 1 representative from each of the 4 cochlear implant companies: Advanced Bionics UK Ltd, Cochlear Europe Ltd, MED-EL UK Ltd, and Oticon Medical Ltd.

We have not established an independent data monitoring committee, as this is not a clinical trial, and it is not a requirement of the funder. The funder may observe, monitor, and inspect the delivery of the project and reserves the right to externally evaluate any aspect of the project and its outputs. The funder may need to allow members of The Health Foundation Research Directorate to inspect anonymized records and data, including recordings and transcripts of interviews with patients and others.

#### Patient and Public Involvement

The project team has a strong commitment to patient and public involvement, and a member of the project team is a service user (CR). Local and national publicity (through the website, Twitter, presentations to National Cochlear Implant Users’ Association, newsletter articles, letters, emails, and Yahoo group) has already invited help in designing the project. Several people using cochlear implants have trialed the CHOICE website and the hearing check before its release and have provided feedback in writing and focus groups.

A risk assessment was approved by the University of Southampton Faculty of Engineering and the Environment on May 15, 2018 (FEERA 15927).

### Data Management

The data will be managed according to the University of Southampton Research Data Management Policy. The study’s data management plan and data protection impact assessment are available upon request. Deidentified data will be kept at the University of Southampton for at least 10 years. If patients decide to stop using CHOICE, we will keep the information we have collected thus far unless participants request that it be deleted. It will not be possible to delete data if they have already been anonymized. Individual cochlear implant centers will retain their own clinical patient data according to local policies.

Regarding evaluation data, only deidentified data will be provided to the independent evaluator, who will handle and store this in accordance with the agreements that are put in place at each site. Wessex AHSN will ensure that the data are handled in line with NHS standards, including data collection, code of practice, and information governance. The AHSN computer network is a private cloud-based system compliant with ISO 27001 and approved under the NHS Information Governance Toolkit. The cloud servers are based in the United Kingdom.

The retention schedule for data collected by Wessex AHSN is as follows:

- Audio recordings will be kept until the publication of the evaluation report (July 2021) and then destroyed.
- All other data, including transcriptions of the audio recordings, will be kept until 12 months after publication of the evaluation report (July 2022) and then securely transferred to the University of Southampton (under the control of the chief investigator) to be retained until 10 years after the study conclusion.

### Ethics and Dissemination

Ethical approval was received in November 2018 from the South Central–Hampshire A research ethics committee (REC reference 18/SC/0658; IRAS project ID 242575), Health Research Authority, and Health and Care Research Wales.

#### Confidentiality

Personal and sensitive personal data will be entered into the web app by the patient. The patient will consent to data sharing. The data will be encrypted before transfer. At the close of the project or before, the data will be deidentified (personal data removed). We cannot guarantee anonymity as adults with cochlear implants are still rare in the general population (approximately 0.01% of the UK population or approximately 1 in 10,000 people).

Interviews (with staff and patients) will be audio recorded using an encrypted dictaphone and transcribed. Any used names will be removed after transcription. Data relating to individuals will not be linked together; that is, individual interviews and individual R-Outcomes data will not be linked. The findings will be linked through a synthesis process at the aggregate level. Safety monitoring and reporting of adverse events will occur according to the requirements of the local and national ethics committees, with full support from the sponsor.

#### Dissemination

The results will be presented locally, nationally, and internationally. Dissemination will include but not be limited to peer-reviewed publications both on the web and in print, conference and meeting presentations, posters, newsletter articles, website reports, and social media. To inform people with cochlear implants of the results, information will be sent to the National Cochlear Implant Users’ Association and other patient groups and the University of Southampton Auditory Implant Service patient newsletter. We have budgeted for our academic publication of clinical results to be gold open access. The results of this evaluation will be published in a report by Wessex AHSN.

## Results

As of July 2021, the trial is closed, and all data collection is complete. The evaluation report is expected to be published in December 2021, and the research data have not yet been analyzed.

## Discussion

### Limitations

A total of 7 sites agreed to participate in the implementation and evaluation of CHOICE. These sites are mostly larger adult cochlear implant centers in England. Sites were self-selected: those participating were the centers that expressed interest in taking part. This means that it is unlikely that these centers are representative of all UK adult cochlear implant centers; they are likely to be more willing to innovate. Given that data collection will commence as soon as centers and patients join CHOICE, there will be variable periods of follow-up.

We expect significant effect modification in subgroups (eg, by age, gender, cochlear implant center, and other demographic factors). Assessing and reporting effect modifications may help identify a subset of patients who would not benefit from remote care. We attempted to control for confounding factors by collecting the demographic and digital readiness data. However, it is possible that there are confounders that remain unaccounted for; for example, we will not collect data on mental health and social support or the impact of the COVID-19 pandemic. It is likely that the concurrent COVID-19 pandemic will be the largest confounding factor in the data. In addition, the coincidental launch of a manufacturer-led remote care pathway (Cochlear Remote Check) for patients with some devices is likely to confound the results. The nature of recruitment for this study (cochlear implant center choosing to be involved and patient choosing to take part) means that there is likely to be a significant bias. Patients who choose to take part in a trial of remote care may not be representative of the broader population of people with cochlear implants. As recruitment is performed via patient and clinic choice, it is not valid to have a control group of people who do not follow a remote care pathway.

We are aiming for 6 months of follow-up data. This may be insufficient to highlight the benefits and limitations of remote care, especially in the climate of change because of the COVID-19 pandemic. In addition, as patients are encouraged to register for CHOICE at any point, there may only be a very short experience of using CHOICE by the end of data collection for many people.

The PAM may not be very sensitive to changes in the empowerment of people using cochlear implants because of its medical perspective. Given that this is the first time there has been a large-scale rollout of a remote care model for cochlear implants, we do not know how many people will participate. Low patient numbers and dropouts are likely to affect the quality of the results, although reporting them will provide important information on the success of the implementation. Patients who discontinue the use of CHOICE will be asked to provide a reason for their withdrawal.

### Conclusions

This project will present the results and learning**s** from the first scale up of a remote care pathway for adults with cochlear implants in the United Kingdom.

## Appendix

Multimedia Appendix 1 Specification for quantitative activity information and source of data (services). Column 2 relates each outcome to a research question.

## Abbreviations

AHSN: Academic Health Science Network
CHOICE: cochlear implant home care
CI-EMP: Cochlear Implant Empowerment Scale
DCE: discrete choice experiment
EAG: evaluation advisory group
EQ-5D-5L: EuroQoL 5-Dimension 5-Level
HUI3: Health Utilities Index Mark 3
NASSS: nonadoption, abandonment, scale-up, spread, and sustainability
NHS: National Health Service
PAM: Patient Activation Measure
PM: project manager
SG: steering group
UK: United Kingdom
