# Supplementary material for: Telemedicine for Adults With Cochlear Implants in the United Kingdom (CHOICE): Protocol for a Prospective Interventional Multisite Study
Source: JMIR Res Protoc. 2022 Apr 13;11(4):e27207. doi: 10.2196/27207 (PMC9047741; doi:10.2196/27207)
Supplement: Multimedia Appendix 1 [file resprot_v11i4e27207_app1.doc]

## Appendix 1. Specification for quantitative activity information and source of data (services). Column 2 relates each outcome to a Research Question (RQ)

| **Outcome** | **RQ** | **Detail** | **Measures** | **Source of data** | **Timepoint** |
| --- | --- | --- | --- | --- | --- |
| Spread: remote care tool is embedded in routine practice | 3 | We would expect to see the percentage of total caseload recommended for remote care increasing over the duration of the project. | Total caseload of cochlear implant users (one year or more post-implant), by quarter  Number of users recommended to register on CHOICE, by quarter  Number of patients changing back to old pathway, by quarter  Number of users registered on CHOICE, by quarter  Actions arising from use of the remote care package, e.g. orders for replacement parts  Number of staff registered CHOICE, by quarter  Clinics wanting to stop offering remote pathway  Patients wanting to continue remote care after the end of the evaluation  % clinics wanting to participate  Patients using the remote care pathway, as a percentage of the total clinic caseload | Clinic or Trust Patient Administration System (PAS) system  CHOICE registration data | At start of evaluation, and then every 6 months, beginning 3 months after CHOICE launch |
| Balancing measures | 3 |  | Contacts from patients having difficulty with remote tools  Additional appointments to train in remote care  Additional appointments for patients concerned about results from remote tools  Clinician caseload ratio (patients with problems: straightforward patients)  Number of patients who register to use the tool but do not log in subsequently | Clinic or Trust PAS system  CHOICE registration data | Throughout |
| Equity of access | 6 | It is hoped that more people with cochlear implants will access follow-up care if they are given a remote care option. This may particularly apply to those who live a long distance from the clinic. Geo-mapping will enable us to locate all clinic attendees – to maintain anonymity, a patient’s partial postcode will be supplied to the AHSN. | cochlear implant sites are to provide:   - Postcodes (shortened to outgoing postcode only, E.g. SO16 2AP will be shortened to SO16) of all those on the clinic caseload (A row listing per patient is recommended), with supporting fields to indicate:   - A person care pathway (either remote care or traditional pathway)   - Date of CHOICE uptake - For all outpatient appointments 36 months before launch of CHOICE and until December 2019, the patients outgoing postcode (e.g. SO16), the clinic outcome (attended or DNA or cancelled) and the reason for not attending clinic appointments (e.g. declined). | Clinic or Trust PAS system | At start of evaluation, and then every 6 months, beginning 3 months after CHOICE launch |
| Change in use of out-patient appointments  Reduction in number of out-patient appointments (additional to pre-planned review appointments, and after one year post implant), following implementation of the tool | 8 | It is expected that post-implementation of the remote care tool, out-patient appointments will be offered on a request basis according to need rather than on a pre-planned basis. We will need to understand the activity of each clinic before implementation of the remote care option and afterwards (what happens to the numbers of out-patient appointments, who initiates them and what for)  The out-patient appointment may be provided by a different member of staff (e.g. technician rather than clinician) if the reason for the appointment is known in advance through using the tool. | For each cochlear implant clinic:   - Total number of outpatient appointments completed by the clinic, by month, and by care pathway (either remote care or traditional pathway) for 36 months before rolling out the remote care system, and until the end of Oct 2019, including Did Not Attend rate. Outpatient activity associated with care of a patient in their first year of having a cochlear implant should be excluded. Data to also include type of appointment to be provided if available (e.g. routine check, problem (urgent or emergency appointment), technical appointment (e.g. upgrade)   For each user recommended and taking up care using the remote care pathway:   - date registered on the on-line tool - number of clinic appointments and contacts by month for past 36 months (or maximum period of time if user has had their implant less than 36 months)and end of December 2019 Appointments associated with the user’s first year of care should be excluded. - Field to show clinic initiated or user initiated appointments (and which staff type if clinic initiated) - reason for out-patient appointment - Role of professional who saw the person (e.g. technician, clinician, etc.) | Clinic or Trust PAS system | At start of evaluation, and then every 6 months beginning 3 months after CHOICE launch |
| Improved use of resources | 8 | What is the costing model for remote care during the project?  Aggregate data on number of out-patient appointments pre- and post-implementation of remote care pathway at each site should show a reduction in out-patient appointments (excluding those that are required as part of routine care) | - Included as part of the data in dataset 1 | Clinic or Trust PAS system | At start of evaluation, and then every 6 months beginning 3 months after CHOICE launch |
| Possible workforce changes | 8 | This detail may be revealed in the qualitative work but should be quantified where possible | Staffing complement (WTE) and job roles at time of implementing remote care pathway. To be updated by the clinic manager throughout the project to note any changes that respond to implementation of the pathway. A template is suggested, below. | Clinic or Trust PAS system | At start of evaluation, and then every 6 months, beginning 3 months after CHOICE launch |

**Sample Workforce every 6 months return template**

| **Role description** | **Staff grade (e.g. Agenda for Change (AfC) band 6)** | **Number of Whole Time Euuivalent (WTE)** | **Have the responsibilities of this role changed in the past quarter, as a result of CHOICE?** |
| --- | --- | --- | --- |
|  |  |  |  |
